# Supplementary material for: Oncological and Functional Outcomes of Hemi-Ablation Versus Focal Ablation for Localized Prostate Cancer Using Irreversible Electroporation
Source: Cancers (Basel). 2025 Jun 22;17(13):2084. doi: 10.3390/cancers17132084 (PMC12248562; doi:10.3390/cancers17132084)
Supplement: Supplementary file 1 [file cancers-17-02084-s001.zip › supplemental table S1.pdf]

Supplemental Table S1: The change of biopsy results after treatment (A: focal ablation group B: hemi-ablation group)

A

ISUP** / ISUP*	Negative	1	2	3	4	5	Total
1	5	5	4	0	0	1	15
2	5	4	1	1	1	0	12
3	0	0	0	0	0	1	1
4	0	0	1	0	0	0	1
Total	10	9	6	1	1	2	29

B

ISUP** / ISUP*	Negative	1	2	3	4	Total
1	17	6	1	1	0	25
2	18	5	1	1	1	26
3	5	0	0	0	0	5
Total	40	11	2	2	1	56

ISUP: International Society of Urological Pathology

ISUP*: ISUP group in the baseline biopsy

ISUP**: ISUP group in the repeat biopsy
