# Supplementary material for: Oncological and Functional Outcomes of Hemi-Ablation Versus Focal Ablation for Localized Prostate Cancer Using Irreversible Electroporation
Source: Cancers (Basel). 2025 Jun 22;17(13):2084. doi: 10.3390/cancers17132084 (PMC12248562; doi:10.3390/cancers17132084)
Supplement: Supplementary file 1 [file cancers-17-02084-s001.zip › supplemental table S2.pdf]

Supplemental Table S2: The characteristics of patients with/without repeat prostate biopsy

| | With repeat biopsy (n=94) (median, IQR) | Without repeat biopsy (n=12) (median, IQR) | P-value |
|---|---|---|---|
| Age (years) | 70 (65-74) | 72.5 (68-74.8) | 0.466 |
| PSA (ng/ml) | 7.7 (5.5-10.2) | 6.8 (2.3-8.5) | 0.408 |
| Prostate Volume (ml) | 45 (30-60) | 32.5 (6.8-49) | 0.093 |
| ISUP | | | 0.509 |
| 1 | 43 | 7 | |
| 2 | 42 | 4 | |
| 3 | 8 | 1 | |
| 4 | 1 | 0 | |

PSA: prostate specific antigen

IQR: interquartile range

ISUP: International Society of Urological Pathology
