# Supplementary material for: Oncological and Functional Outcomes of Hemi-Ablation Versus Focal Ablation for Localized Prostate Cancer Using Irreversible Electroporation
Source: Cancers (Basel). 2025 Jun 22;17(13):2084. doi: 10.3390/cancers17132084 (PMC12248562; doi:10.3390/cancers17132084)
Supplement: Supplementary file 1 [file cancers-17-02084-s001.zip › supplemental table S3.pdf]

Supplemental Table 3: The change of quality of life measured with IPSS and IIEF

Variable	Focal ablation (median, IQR)	Hemi-ablation (median, IQR)	P-value
IPSS			
Baselines	5 (2.5-10.5)	7 (2-12)	0.525
At 3 months	5 (3-8)	6 (4-10)	0.295
At 6 months	5 (2-8)	5 (3.8-8.3)	0.278
At 12 months	4 (1-8)	4 (2-6.3)	0.516
At 18 months	2.5 (1-9)	7 (5-10)	0.107
At 24 months	3 (1.5-10)	5.5 (4.3-9.3)	0.423
IIEF			
Baselines	11 (5-19)	16 (5-20)	0.360
At 3 months	14 (3.3-18.5)	7 (2.5-14)	0.138
At 6 months	12 (7-17)	6 (4-17.5)	0.353
At 12 months	14.5 (4.5-20.3)	7 (4.5-18)	0.554
At 18 months	14 (8-18)	9 (3-19.5)	0.456
At 24 months	15 (6.5-20)	10.5 (5-16)	0.388

IPSS: International Prostate Symptom Score

IIEF: International Index of Erectile Function

IQR: interquartile range
